# Progression of CIN1/LSIL HPV Persistent of the Cervix: Actual Progression or CIN3 Coexistence

**DOI:** 10.1155/2021/6627531

**Published:** 2021-03-09

**Authors:** Maria Teresa Bruno, Nazario Cassaro, Francesca Bica, Sara Boemi

**Affiliations:** ^1^Department of General Surgery and Medical Surgery Specialties, Gynecological Clinic, University of Catania, Italy; ^2^Humanitas Medical Care Catania, Catania, Italy

## Abstract

**Objective:**

The natural history of the CIN1 lesions is characterized by an elevated rate of spontaneous regression (80%), some authors recognize a capacity to progress to HSIL in 10% of cases, and other authors do not recognize the capacity of progression of LSIL (CIN1). This study was aimed to evaluate the incidence of progression to HSIL (CIN3) in women with a histological diagnosis of LSIL (CIN1). Furthermore, to this end, we studied the histological outcomes of cone specimens collected by the LEEP.

**Methods:**

All the data were retrospectively analyzed. All participants underwent a follow-up of 4 years, during which each woman underwent an HPV test and genotyping, cervical cytological sampling, or biopsy every six months. The endpoint was the histological confirmation of CIN3 lesions in any moment during follow-up.

**Results:**

Progression to CIN3 occurred in 7 cases (1,5%). Analyzing the histological exams of the cones of the 7 cases that progressed to CIN3, we found the coexistence of CIN1 and CIN3 lesions in all cases.

**Conclusion:**

After 4 years of follow-up, only 1.5% (7/475) of the women with LSIL developed CIN3, all within the first two years of follow-up, and were immediately treated. The most likely explanations for “progression” from LSIL to HSIL are (1) actual progression, (2) underdiagnosis of HSIL on initial biopsy, (3) overdiagnosis of HSIL on follow-up biopsy/cone, and (4) CIN3 arose de novo. Analyzing the histological exams of the cones of the 7 cases that progressed to high-grade, we found the coexistence of CIN1 and CIN3 lesions in all cases. Some recent studies have shown that a viral genotype corresponds to different lesions in the same cervix; therefore, CIN1 coexisting with CIN3 does not always indicate progression of CIN1. Other authors have doubted the capacity of LSIL to progress.

## 1. Introduction

There is still much debate concerning the management of patients with LSIL (CIN1) of the uterine cervix, defined as the transient expression of a self-limited human papillomavirus (HPV) infection [1]. The amount of scientific evidence published over the last few years regarding the risk of progression/regression of this lesion has conditioned the diagnostic and therapeutic orientation of Italian and international scientific societies [2, 3]. The guidelines currently available offer the possibility of treatment or continuation of follow-ups if LSIL (CIN1) persists for at least two years [4].

The natural history of the CIN1 lesions is characterized by an elevated rate of spontaneous regression (80%) [5, 6], some authors recognize a capacity to progress to HSIL in 10% of cases [7, 8], and other authors do not recognize the capacity of progression of LSIL (CIN1) [9].

In light of these considerations, this study was aimed to evaluate the incidence of progression to HSIL (CIN3) in women with a histological diagnosis of LSIL (CIN1) and the correlation with the genotype as well as the type of viral infection, with six monthly follow-ups for 4 years. Furthermore, to this end, we studied the histological outcomes of cone specimens collected by the loop electrosurgical excision procedure (LEEP).

## 2. Materials and Methods

We enrolled 751 women at the outpatient clinic of Colposcopy and Cervico-vaginal Pathology at the University of Catania, Italy, with a cytological diagnosis of LSIL (CIN1).

The first participants were enrolled in March 2014.

Patients' age was from 24 to 49 years, with an average of 29 ± 8.3 years and a median of 33 years.

Inclusion criteria are as follows:

- Positive at the cytological and histological exam for LSIL/CIN1
- Positive HPV test
- Completed 4 years of FU

Women were not enrolled in this study if they were pregnant, with a diagnosis of or under therapy for previous cervical dysplasia, immune-depressed, or with infections caused by human immunodeficiency virus (HIV-positive).

All the data of the women in our database who met the inclusion criteria were retrospectively analyzed in an observational cohort study.

Only 475 patients met the inclusion criteria; their clinical data was gathered regarding sexual and daily habits that could facilitate virus acquisition.

All the participants underwent colposcopy guided biopsy of suspected areas after an anomalous cervical cytology examination and successive colposcopy, according to current guidelines [3, 4].

All participants underwent a follow-up of 4 years, during which each woman underwent an HPV test and genotyping, cervical cytological sampling (Bethesda system), and all the participants with positive colposcopy or viral persistence underwent biopsy every six months. The endpoint was the histological confirmation of CIN3 lesions in any moment during follow-up.

The women who showed progression to CIN3 underwent large loop excision of the transformation zone (LEEP).

Histologic evaluation was performed with specimens collected by a colposcopy-directed biopsy and/or cone specimens collected by the loop excision procedure. Chronological data of follow-ups were compiled and analyzed for regression, persistence, and progression of the lesions over time. Regression is defined as CIN 1 lesions that return to normal cytology. Persistence is defined as CIN 1 lesions demonstrated by biopsy. Progression is defined as histologically confirmed CIN3. In this retrospective study, all the histological diagnoses were reviewed.

The search for viral DNA (HPV-test) was carried out using PCR after the extraction of the cervical sample using Thin-prep. The automated DNA extraction was carried out on the NucliSenseasyMAG system (bioMérieux SA, Marcy l'Etoile, France) following the manufacturer's HPV 1.1 protocol. Amplification of HPV DNA was accomplished by HPV-HS Bio (AB Analiticas.r.l, Padova, Italy) nested polymerase chain reaction (PCR) for the detection of HPV-DNA sequences within the L1 ORF, according to the manufacturer's recommendations.

HPV typing was carried out with specific probes for the most frequent HPV types (HPV-type, AB Analitica s.r.l., Padova, Italy). HPV typing allows the identification of 11 LR genotypes (6, 11, 40, 42, 43, 44, 54, 61, 70, 72, and 81) and 18 HR genotypes (16, 18, 26, 31, 33, 35, 39, 45, 51, 52, 53, 56, 58, 59, 66, 68, 73, and 82). Samples that were positive by nested PCR but negative in reverse line blot for any of these types were considered as undetermined HPV. The cervical swab for the HPV test was taken from the endocervical canal and the transformation zone.

We classified HPV infection in transient if the second genotyping shows a different genotype, persistent if the second sample shows the same genotype after 6 months or 1 year from the first, clearance if two consecutive samples of DNA HPV are negative at an interval of about 6 months after a positive sample. Viral clearance does not always coincide with regression of the lesion; thus, the women underwent HPV (viral clearance) and cytological samples or biopsy. After 2 negative cytological samples and a negative HPV test, women reenter screening (viral clearance).

Moreover, a single HPV infection is characterized by the presence of a single genotype, multiple HPV infections from the concomitant presence of more genotypes; in this case, the causality is attributed based on the finding of the same HPV type of the previous samples.

### 2.1. Ethical Approval

The study was conducted in accordance with the 1975 declaration of Helsinki. The investigations were conducted through the retrospective review of the medical database; the protocol was notified to the Catania1 Ethics Committee of the Catania Polyclinic, according to the AIFA law of 20 March 2008. Furthermore, the consent of the study participants was deemed unnecessary as the study only concerned the retrospective review of the medical database.

### 2.2. Statistical Analysis

Statistical analysis of the data was made using the software package SPSS 15.0 (SPSS Inc.; Chicago, IL, USA). Descriptive statistics were expressed as frequency, arithmetic mean, standard deviation (S.D.), and percentages. The analysis of the data was made using the *χ*^2^ test, and, when appropriate, Fisher's exact test was used to calculate the significance (*p* value) of the difference between groups.

## 3. Results

At the histological examination, carried out after biopsy, 511/626 patients (81,7%) had one low/grade precancerous lesion (LSIL/CIN 1); 115 (18.3%) patients had a negative histological diagnosis. The 511 CIN1 positive women were given an HPV test; of these, 36 samples (7%) were found to be inadequate; these patients were excluded from the study. Thus, the study group was composed of 475 women who were CIN1 positive at the histological exam and HPV positive and completed the four years of follow-up.

Viral genotyping was positive for infections from a single HPV genotype for 180/475 patients (37.9%), and 295/475 patients (62.1%) were, instead, positive for multiple infections (Table 1). We had 414 cases (87.1%) of transient infection and 61 cases (12.9%) of persistent infection.

All the participants (475 women) completed the follow-up period (48 months), and 1.5% (7/475) developed CIN3 and represented 11.5% (7/61) of the women who had persistent HPV infection.

We found 61 (12.9%) persistent infections and 414 (87.1%) transient infections (Table 1).

Of the transient infections, 251 were multiple and 163 were single; in almost all of the cases, viral clearance occurred within 18 months and regression of the lesion within 24 months; in 9% of the cases, infection was transient from papillomavirus even if the CIN1 lesion disappeared. The rate of regression for histologically confirmed CIN1 lesions was always elevated. The rate at 12 months was 54,8% and at 24 months 35,9%. Overall transient infections histologically regressed in 100% of the cases within the 4 years of follow-up. In transient infections, both single and multiple, genotypes 16, 54, 58, and 31, were the most frequent.

**Figure 1:**
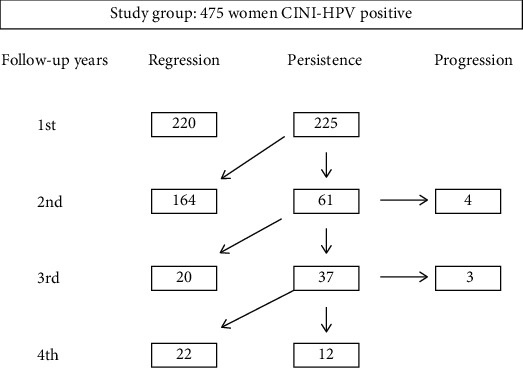
Flow Chart of the regression, persistence, and progression histological during 4 years of follow-up in the study group.

We had 61 cases of persistent HPV infection, 17 single infections, and 44 multiple infections, in our study. In this group, viral and histological lesion clearance occurred in 43 cases (33 multiple and 10 single), with an average persistence of 2.5 years, and progression to CIN3 in 7 cases (5 cases of single infection and 2 cases of multiple infection). Progression occurred in the first 24-36 months of follow-up (Figure 1).

The most frequent genotypes in the persistent infection group were HPV 16, HPV 31, and HPV 33. To date, 12 cases have long-term persistence. Analyzing the histological exams of the cones of the 7 cases that progressed to CIN3, we found the coexistence of CIN1 and CIN3 lesions in all cases (Table 2).

## 4. Discussion

The diagnosis and prevention of cervicocarcinoma are based on the presupposition of Richard [10] that preneoplastic lesions evolve slowly in time and progress by means of a spectrum of histological differentiation that goes from grade I (CIN1 or mild epithelial dysplasia), over grade II (CIN2 or moderate dysplasia) to grade III (CIN3 or severe dysplasia), and then to invasive carcinoma.

An important role is played by a woman's immune system and by the presence of cofactors such as smoking, use of oral contraceptives, and genital herpes infections. Furthermore, it has been demonstrated that the natural history of the evolution of CIN depends on the type of HPV that infects the cervix [11] and the type of infection.

Some authors [12–14] have shown that the prevalence of multiple HPV infections is elevated in cases of mild dysplasia, decreases in CIN2 and in CIN3 to disappear in neoplasia, while the prevalence of infection by a single genotype is less elevated in CIN1, increasing in severe dysplasia and carcinoma. In our cases of CIN1 lesions, multiple infections were 62.1% of the cases (295/475), while single infections were 37.8% of the cases (180/475). Moreover, most HPV infections were transient (87.1% %), while the cases of persistence were 12.9%. Histological regression, as LSIL is the biological manifestation of one infection produced by HPV, is reached with viral clearance, typical of transient lesions. Generally, viral clearance occurs in 12-18 months, followed by histological regression. All the CIN1 lesions caused by transient HPV infections achieved clearance and histological regression.

The cases of persistent HPV infection were 12.9% (61/475), 68,9% (42/61) of these cases reached clinical recovery with viral clearance, persistence had an average duration of 2.5 years, 19.6% (12/61) persist to date, without alterations of the histological state of CIN1, and 11.5% (7/61) of cases had progression; in 7 cases to CIN3, all these lesions occurred during the first 36 months of F0ll0w-up.

Overall, 82.1% of the women with CIN1 regressed within 2 years, while 1.5% progressed to a high-grade lesion within 3 years.

Moreover, 19.6% (12/61) of persistent infections were not able to eliminate the virus in 4 years.

The fact that our study sample is made up of women already at baseline with low-grade lesion and positive for HPV infection does not allow us to know the time between the first detection of HPV infection and the first detection of cytological abnormalities. We can say that from the observation of the woman with LSIL to the onset of the high-grade lesion, the average of 36 months has elapsed. This finding is consistent with previous studies that reported the onset of HSIL within 3 years of the first detection of HPV infection. Even authors [11, 15] assert that the time between the first detection of HPV and the first detection of cytological abnormalities is similar for all grades of CIN.

Transient HPV infections were clinically irrelevant with a high percentage of viral clearance and histological regression, while cases of viral persistence are those that clinicians have to be interested in: they are nearly all linked to a single genotype; in fact, cases of persistence of 2 or more genotypes are very rare. It is known that the persistence of specific viral genotypes is associated with a greater risk of developing high-grade lesions. In particular, persistent HPV 16 infection is known as the most significant prognostic factor in the progression of cervical lesions.

In our precedent study [16], the presence of HPV16 genotype was associated with a risk 5 times greater of developing a high-grade lesion (OR = 4.62; 95 CI: 3,13-6,82).

The most represented genotypes in persistent infections with progression are HPV 16 and HPV 31, the most frequent in Italy and Europe [17], while HPV 16 and HPV 33 are associated with the highest risk in the USA. These results are in agreement with other studies as reported by Schiffman [18], PATRICIA Study [19], and VIVIANE Study [20]. In our analysis, HPV 31 indicated a risk of CIN3 in the ≥25 year age group, which was second only to HPV 16.

Recent data from the control arm of the FUTURE I study reported a persistence of 12 months or more, and the highest risk of progression to CIN2+ for HPV16 and HPV33 [21].

The women with progression were, on average, 32 years old and had one single persistent infection with one of the following genotypes: HPV 16, HPV 31, or HPV 33. The low rate of subsequent CIN 3 suggests that follow-up time, rather than immediate ablation or resection of the transformation zone, is appropriate in these women. Excisional treatments are associated with physical, psychological, and obstetric morbidity [22] and can have a negative impact on sexual function [23]. Overall, in our study, we found a low rate of progression towards HSIL (1.5%). After 4 years of follow-up, only 1.5% (7/475) of the women with LSIL developed CIN3, representing 11.5% (7/61) of the women with persistent HPV infection. Ostör indicated that the probability of progression from CIN1 to CIN3 was ∼10%, in the study of Pretorius, the risk of CIN3 was 1.9%, less than the study of Ostör, but very near our results. The rate of CIN3 following an LSIL diagnosis is so sufficiently low (1,5%) that any follow-up diagnosis of CIN3 in this circumstance would prompt both the review of the original biopsy and the outcome diagnosis of preneoplastic lesion.

The most likely explanations for “progression” from LSIL to HSIL are (1) actual progression, (2) underdiagnosis of HSIL on initial biopsy, (3) overdiagnosis of HSIL on follow-up biopsy/cone, and (4) CIN3 arose de novo. There is a significant risk that an HSIL diagnosis following an LSIL biopsy is a diagnostic error and should be reviewed by an additional observer. Less likely is that the initial LSIL biopsy is an undercalled HSIL (CIN3). Analyzing the histological exams of the cones of the 7 cases that progressed to high-grade, we found the coexistence of CIN1 and CIN3 lesions in all cases.

We asked ourselves if CIN1 is the same lesion for which the woman underwent follow-up and that it remained the same and thus did not progress, if CIN3 has always been present and not diagnosed or if it occurred “de novo” over about 6 months. Park [24] demonstrated how HPV infection can cause CIN1; a successive infection, also of a different genotype, can cause a high-grade lesion not correlated with low-grade CIN; thus, CIN1 and CIN3 lesions could be caused by different HPV in the same cervix and therefore originate from different cellular clones with different clinical outcomes. A recent study [25] showed that a viral genotype corresponds to different lesions in the same cervix; therefore, CIN1 coexisting with CIN3 does not always indicate progression of CIN1.

In our cases, we had a rapid development of high-grade lesions; in fact, the cases of CIN3 and carcinoma occurred within 24-36 months of follow-up to which the women were undergoing for persistent HPV CIN1. Other authors have doubted the capacity of LSIL to progress [9].

Today, it is accepted that CIN3 can arise de novo, rather than be the result of a continuum of CIN1. It is known that CIN1 is due to a viral cytopathic effect and not necessarily the onset of the progression to cancer. Lityens [26] show that CIN3 lesions are rarely preceded by a CIN1 lesion and the presence of LSIL does not determine the risk of consequent HSIL.

Therefore, from the analysis of the data, we can say that it is not the presence of CIN1 that determines the risk of a successive CIN3, but the diagnosis of persistent HPV 16, 31, 33, or 18 infection. This knowledge would avoid carrying out intensive follow-ups and unnecessary treatment (all the women with CIN1) and the missing diagnosis at biopsy of CIN3, arose de novo, coexisting with CIN1.

## Figures and Tables

**Table 1:** Percentage of regression, persistence, and progression in the study group.

	No. cases	Regression	Persistence	Progression
Transient HPV infection				
Single infection	163	163	0	0
Multiple infection	251	251	0	0
Total	414 (87,1%)	414 (100%)	0	0
Persistent HPV infection				
Single infection	17	10 (58,5%)	2 (11,7%)	5 (29,4%)
Multiple infection	44	32 (72,7%)	10 (22,7%)	2 (4,5%)
Total	61 (12,9%)	42 (67,7%)	12 (19,6%)	7 (11,5%)
	475 (100%)	456 (96%)	12 (2,5%)	7 (1,5%)

**Table 2:** Analyzing the histological exams of the cones of the 6 cases that progressed to CIN3, we found the coexistence of CIN1 and CIN3 lesions in all cases.

Patients	Age	Follow-up years				HPV genotypes	Biopsy	Histology of LEEP
		1st	2nd	3rd	4th			
1	25	CIN1	CIN1	CIN3		18, 39	CIN3	CIN1- CIN3
2	37	CIN1	CIN1	CIN3		16	CIN3	CIN1-CIN3
3	31	CIN1	CIN3			33	CIN3	CIN1-CIN3
4	34	CIN1	CIN3			31	CIN3	CIN1-CIN3
5	42	CIN1	CIN1	CIN3		16	CIN3	CIN1-CIN3
6	40	CIN1	CIN3			31	CIN3	CIN1-CIN3
7	32	CIN1	CIN3			16, 33	CIN3	CIN1-CIN3

Abbreviations: CIN1: cervical intraepithelial neoplasia grade 1; CIN3: cervical intraepithelial neoplasia grade 3; LEEP: Loop Electrosurgical Excision Procedure.

## Data Availability

The data used to support the findings of this study are available from the corresponding author upon request.

## Abbreviations

LSIL: Low-grade squamous intraepithelial lesion
HSIL: High-grade squamous intraepithelial lesion
CIN1: Cervical intraepithelial neoplasia grade 1
CIN3: Cervical intraepithelial neoplasia grade 3
HR-HPV: High-risk HPV.
